# New insights into the integrative effects of resistance training at moderate altitude on systemic inflammation

**DOI:** 10.1007/s00421-025-05842-x

**Published:** 2025-06-07

**Authors:** S. Pérez-Regalado, J. Leon, P. Padial, C. Benavente, F. Almeida, J. Bonitch-Gongora, B. Feriche

**Affiliations:** 1 Department of Physical Education and Sport, Faculty of Sport Sciences, University of Granada, Granada, Spain; 2 Clinical Management Unit of Digestive System, San Cecilio Hospital, Ibs.GRANADA, Granada, Spain; 3 Sport and Health University Research Institute (iMUDS), University of Granada, Granada, Spain

**Keywords:** Hypoxia, Resistance training, Altitude, Inflammation, Cytokines

## Abstract

**Purpose:**

To determine the effect of intermittent terrestrial hypobaric hypoxia (HH) exposure on stress and inflammatory biomarkers following a resistance training (R_T_) program.

**Methods:**

Twenty trained males completed an 8-week R_T_ program (3 sessions/week) under HH (2320 m asl) or normoxia (N, 690 m asl). Before and after the R_T_, circulating stress biomarkers (calcium, inorganic phosphate, creatine kinase [CK], total antioxidant capacity [TAC]), inflammation (tumour necrosis factor-alpha [TNF-α]), interleukin 10 (IL-10), vascular endothelial growth factor and heat shock protein 70 (HSP70) were analyzed by immunology multiplex assay and ELISA. Moreover, maximal strength to back squat (1RM-SQ) and squat jump (SJ) performance were measured.

**Results:**

The results revealed that, compared with N, the HH group exhibited a large increase in 1RM-SQ and SJ (all ES > 0.99; *p* < 0.041) outcomes. IL-10 and TNF-α levels increased in HH more and faster than N (all ES > 1.35; *p* < 0.003), returning to baseline following the R_T_. Circulating HSP70 revealed a similar trend, although remaining elevated in HH after the program (all ES > 1.106; *p* < 0.029). HSP70 in HH explained ~ 44% of TNF-α variance (*p* < 0.001). In addition, the R_T_ program in HH induced greater decreases in TAC and CK than N (all ES < − 0.95; *p* < 0.05).

**Conclusions:**

Findings highlight the potential role of moderate altitude in long-term R_T_ for inducing greater stress while maintaining the inflammatory balance, crucial for muscle adaptations in young males. Consequently, HH condition revealed an additional benefit in the contractile and explosive muscle strength development.

**Supplementary Information:**

The online version contains supplementary material available at 10.1007/s00421-025-05842-x.

## Introduction

Skeletal muscle is essential for locomotion and overall health, and its decline can severely impair physical function, negatively affecting both sports performance and daily living tasks (Goodpaster et al. 2006; Metter et al. 2002). The extent of these impairments is largely influenced by muscle quantity and quality (D'Souza et al. 2013). In this sense, resistance training (R_T_) emerges as a well-established strategy for enhancing muscle strength and hypertrophy (Benavente et al. 2023), with substantial evidence supporting its efficacy both in acute (Dankel and Razzano 2020) and long-term interventions (Zhao et al. 2022). In the context of lower limb muscle strength assessment, the vertical jump constitutes a reliable and valid tool for measurement. In particular, the squat jump (SJ) test is widely used to assess skeletal muscle contractile capacity (Petrigna et al. 2019), and outcomes are closely related to inflammatory status (Siqueira et al. 2018). Muscle power, which is strongly dependent on maximal strength enhancement, modulates the SJ performance (Stone et al. 2022). Based on these considerations, recent research has focused on incorporating novel approaches, such as resistance training in hypoxia (RTH), for potentially enhancing muscle adaptations beyond the conventional interventions.

Terrestrial hypobaric hypoxia (HH) represents an environmental stressor that challenges human physiology in several ways. Acute exposure to moderate HH environments (2000–3000 m asl) (Bergeron et al. 2012) has been shown to induce significant physiologic stress (Kim et al. 2021), particularly when combined with resistance exercise, which exacerbates the systemic inflammatory response (Pérez-Regalado 2023; Feriche et al. 2017). A recent meta-analysis (Khalafi et al. 2023a) examined the effect of acute exercise in hypoxia on inflammatory cytokines, IL-10 being the sole one greatly enhanced in hypoxia compared with N. Notably, only one of the three studies that employed resistance exercise in the meta-analysis was conducted at terrestrial HH (Benavente et al. 2021). To date, just two studies have investigated the inflammatory and oxidative stress responses to acute resistance exercise at moderate HH (Pérez-Regalado 2023; Benavente et al. 2021). Particularly, Pérez-Regalado et al. (Pérez-Regalado 2023) reported an elevated secretion of inflammatory cytokines, counterbalanced by a proportional anti-inflammatory response. However, current knowledge about the cumulative effects of prolonged R_T_ programs combined with intermittent HH exposure on systemic inflammatory responses remains limited. To date, only two investigations have explored the chronic adaptations to R_T_ performed under moderate HH (Benavente 2024; Pérez-Regalado et al. 2024). Compared with normoxia (N), changes in metabolic cytokines involved in adipose tissue regulation favoring the HH condition were found (Pérez-Regalado et al. 2024). Moreover, despite no significant differences were found between HH and N regarding the strength outcomes, there was a large effect favoring the HH condition (Benavente 2024). Accordingly, a recent meta-analysis (Benavente and Feriche 2024) suggested only a trivial benefit of R_T_ interventions under intermittent terrestrial or normobaric hypoxia, lasting at least 3 weeks, compared with normoxia (N) in muscle strength gains. However, the variability of the protocols employed may have potentially influenced this outcome.

Evidence in N suggests that 9 weeks of R_T_ (3 days per week; 10–12 reps; 40–80% of 1RM) progressively reduced baseline systemic inflammation in healthy young participants, shifting toward increased anti-inflammatory mediators (Forti et al. 2017). This process involves inflammatory cytokines, such as tumour necrosis factor-alpha (TNF-α) or interleukin-1 beta (IL-1β), among others, both serving as main mediators of muscle damage and apoptosis pathways (Cornish et al. 2020). Conversely, interleukin-6 (IL-6) operates in a stimulus-dependent manner, exhibiting a pro- or anti-inflammatory role (Forti et al. 2017). Immediately after acute exercise, IL-6 is rapidly released, playing a key role in mediating anti-inflammatory responses (e.g., IL-10, IL-1Ra) (Docherty et al. 2022). Likewise, exercise-induced reactive oxygen species (ROS), muscle damage, and metabolic shifts, in addition to muscle hyperthermia (Noble et al. 2008), also activate and release heat shock proteins (HSPs) as required to maintain cellular homeostasis and ensure cell survival. Specifically, the HSP70 modulates the immune system and promotes anti-inflammatory cytokine secretion, such as IL-10, while attenuating muscle damage response (Borges et al. 2012; Liu et al. 1999). Moreover, HSP70 is sensitive to hypoxia, displaying an upregulation associated with hypoxia-inducible factor 1-alpha (HIF1-α)-mediated pathways (Huang et al. 2009), being the precise mechanism involving R_T_ exercise in hypoxia unknown. HSP70 plays an essential part in HIF1-α maturation processes (Sluis et al. 2009), thus contributing to stress tolerance, thereby facilitating cytoprotective effects (Guo et al. 2007). These findings suggest that the modulation of HSP70 in response to combined R_T_ and HH may be a key mechanism in regulating the inflammatory response and enhancing muscle function, warranting further investigation into its combined effects on R_T_ under hypoxic conditions. Inflammation is not merely a transient response following exercise but plays a key modulatory role in initiating and orchestrating long-term adaptations (Bouredji et al. 2022), which would be influenced by the nature of the environmental or exercise stimulus. Understanding how environmental conditions such as HH modulate the inflammatory response to R_T_ is particularly relevant, as hypoxia has been suggested to acutely affect systemic inflammation (Pérez-Regalado 2023), potentially influencing the effectiveness of R_T_ interventions.

Therefore, the present study aims to determine the accumulative effects of a R_T_ program on circulating exercise-induced stress and inflammatory biomarkers under N and moderate HH conditions. This investigation hypothesizes that intermittent HH combined with a long-term R_T_ program will mitigate the “a priori*”* enhanced hypoxia-induced inflammatory response compared with the same exercise in N, ultimately enhancing maximal strength and vertical jump performance.

## Materials and methods

### Design

A longitudinal design with two independent controlled groups and intra- and inter-group measurements was used to analyze the chronic effect of an 8-week R_T_ program on metabolic stress, systemic inflammation, and antioxidant capacity, as well as their influence on muscle strength and explosive performance. The participants were allocated by convenience to the N (690 m asl; age: 22.7 ± 3.37 years; body mass: 72.00 ± 7.70 kg; height: 175.3 ± 4.11 cm; BMI: 23.43 ± 2.51 kg/m^2^) or HH group (2320 m asl; age: 22.8 ± 4.24 years; body mass: 74.03 ± 13.87 kg; height: 177.3 ± 7.40 cm; BMI: 24.09 ± 4.51 kg/m^2^).

### Participants

Twenty Sport Sciences male students volunteered to participate in the study. They were all physically trained and familiarized with the functional R_T_ exercises employed in the study. All participants had experience in R_T_, training at least three times per week for a minimum of the previous year. The participants had no health or muscular disorders and were unacclimated to high altitude (no altitude exposure in the 2 months before the study). All participants lived in N and were exclusively exposed to altitude during the training sessions. The participants were instructed to maintain their habits and regular dietary consumption during the entire study. To standardize post-exercise nutritional intake, they were provided with a protein shake supplement (109 kcal per 30 g of serving; 0.4 g fat, 1.2 g carbohydrate, 25 g protein) 30 min after each training session. This study was approved by the local Ethics Committee (PEIBA: 2212-N-21) and was conducted in accordance with the Declaration of Helsinki and Biomedical Research (14/2007). Informed written consent was obtained from all participants before the beginning of the study.

### Procedure

One week before the start of the R_T_ program, participants engaged in a preparatory session to determine the training load (70% of 1 repetition maximum [1RM]) for each exercise (see for further detail (Benavente 2024)). The relative maximal strength (kg⋅kg^–1^ of body mass) to the back squat (1RM-SQ) exercise was determined through the assessment procedure of the 1RM. Two days before the beginning of the study, participants visited the laboratory for baseline anthropometric measurements (height [Seca 202, Seca Ltd., Hamburg, Germany] and body mass [Tanita TBF-300, Tokyo, Japan]) after fasting since midnight of the previous day. Testing and training sessions were conducted at the same time of the day and in the same training room, at a temperature of ~ 22 °C and ~ 60% humidity for the N condition or ~ 22 °C and ~ 28% humidity for the HH condition. Arterial oxygen saturation (SpO_2_; Wristox 3100; Nonin, Plymouth, MN, USA) was assessed per duplicate before the start of the warm-up of each R_T_ session to test the HH condition (N: 97.64 ± 1.23%, HH: 93.71 ± 1.59%; *p* < 0.001).

### Training program

The participants performed an 8-week R_T_ program on non-consecutive days (3 days per week; 22 sessions in total). The same R_T_ program was used for both environmental conditions. The R_T_ sessions comprised 3 sets of 8–10 repetitions, 90 s rest between sets at 70% 1RM of 6 functional exercises involving the main muscle groups of the body (full-body routine: back squat, deadlift, pull-down, bar row, bench press, and military press). A standardized warm-up of 15 min was completed at the beginning of each training session. The HH group performed the training sessions under terrestrial HH conditions at the High-Performance Centre of Sierra Nevada (2320 m asl, Spain). On every training day, participants were transported by car to the altitude Centre. Arrivals occurred approximately half an hour before the training session started, and they immediately returned to normoxia after completing it.

### Blood measurements

After 2 days of refraining from any exercise, all the participants attended the laboratory 72 h before the first training session (pre-R_T_) under fasted conditions for resting blood sample collection in normoxic conditions. Blood samples were also taken at minutes 5, 10, and 30 after the first (S1) and the last (S22) R_T_ session in the corresponding environmental condition for each group. Circulating variables, such as metabolites (inorganic phosphate [Pi] and calcium [Ca^2+^]), total antioxidant capacity (TAC), and cytokines (IL-10, TNF-α, and HSP70), were determined. Peak values of each studied variable throughout the post-exercise recovery time window were used for analysis. Immediately after the exercise, an antecubital forearm vein was canalized via a catheter and remained permeable using a physiologic saline solution. Two mL of blood before each extraction were discarded to avoid sample dilution. All blood samples were kept in cold conditions after extraction and centrifuged to isolate serum within 4 h at 3000 rpm for 10 min. Finally, several 500-µl aliquots of serum were stored at − 70 °C until use.

Cytokines (IL-10 and TNF-α) were assessed using the Milliplex Human High Sensitivity T Cell Panel (HSTCMAG-28SK) from Sigma-Aldrich (Darmstadt, Germany). The assay sensitivity for cytokines is 0.11–8.17 pg/ml (intra-assay coefficient of variability [CV] < 11.4%, inter-assay CV < 12%). Vascular endothelial growth factor (VEGF) secretion was measured with a human VEGF ELISA kit (Life Technologies, France, #KHG0111). The assay sensitivity for this kit is < 5 pg/ml (intra-assay CV: 8.1%, inter-assay CV: 4.7%). The concentration of HSP70 was measured with an ELISA kit DuoSet IC ELISA kit (R&D Systems; Minneapolis, USA). The assay sensitivity for the HSP70 kit is 2 ng/ml (intra-assay CV: 5.3%, inter-assay CV: 4.5%). TAC was determined using a Total Antioxidant Capacity Assay Kit (MAK187-1KT) from Sigma-Aldrich. The detection range of the kit is 4–20 nmol/µl (intra-assay CV: 9%, inter-assay CV: 9%). Quantitative data were obtained using the Luminex-200 system (Luminex Corporation, Austin, TX, USA), and data analysis was performed with Luminex 100™ IS v2.3 software. Serum Pi and Ca^2+^ were analyzed in a COBAS C-311 instrument from Roche (Indianapolis, IN, USA). The detection range of the kits is 0.62–5.54 mmol/l for Pi and 0.28–4.65 mmol/l for Ca^2+^ (intra-assay CV: < 1.7%, inter-assay CV: < 1.9%). CK activity was analyzed using a COBAS kit from Roche (Indianapolis, USA). The detection range of the kit is 0.3–300 ng/mL (intra-assay CV < 2.9%, inter-assay CV < 6.7%). All procedures followed the manufacturer’s instructions. All analyses were performed in N using the same equipment by specialized staff.

### Lower limb performance

The contractile strength capacity of the lower body was estimated from the change in the SJ height and back squat-1RM tests performed 48 h before (pre-R_T_) and after (post-R_T_) the training period. They both were assessed in the same testing session in this order. After a standard warm-up (5 min aerobic exercise; 1 set × 10 reps of general and specific joint mobility and 2 sets × 5 SJ; 2 min rest), participants performed two valid SJ trials separated by 60 s of recovery. Subjects began from a half squat position (knees and hips flexed at ≈ 90º), with their hands placed on the hips. After 2 s of rest, participants were instructed to jump as high as possible without performing a countermovement nor arms swinging. When an unsuccessful jump occurred, a third attempt was made after an additional minute of rest. All SJ tests were performed on a force plate (Kistler 9281EA, Kistler Instrument AG, Winterthur, Switzerland) connected to the computer software MARS (Measurement, Analysis & Reporting Software). The ground reaction force was recorded at a sampling rate of 1000 Hz. The impulse-momentum approach was used to calculate the instantaneous velocity for each time interval. Jump height was calculated from the take-off velocity. The highest jump was considered for the analysis.

### Statistical analysis

Data are presented as the mean difference ± SD. Data normality assumptions were tested using the Shapiro–Wilk test (*p* < 0.05). We employed the three-sigma rule of thumb method to determine the presence of outliers in the raw data (Bargees and Al-Shuhail 2022).

First, differences between the environmental condition and time on the delta change (Δ) of myokines (IL-10, TNF-α, and HSP70), metabolites (Pi, and Ca^2+^), and TAC following the R_T_ program were interpreted through a two-factor analysis of variance (ANOVA) with a within-group factor (time effect with 2 levels [S1 and S22]) and an inter-group factor (environmental effect with 2 levels [HH vs. N]). Delta change was calculated as the difference (post-exercise – pre-exercise) of the peak values between the last and the first training sessions (ΔS22 vs. S1). Significant main effects and interactions were subsequently analyzed using Bonferroni post hoc tests. Partial eta squared for main effects (η^2^_p_) was obtained from the ANOVA and was interpreted as ≥ 0.01 (small), ≥ 0.06 (medium), and ≥ 0.14 (large) (Cohen 1988).

Second, independent-sample t tests were used to determine the effect of the R_T_ program (absolute delta changes [post-exercise value – pre-exercise value]) between environmental conditions (HH vs. N) for 1RM-SQ and SJ. The IL-10/TNF-α ratio was also evaluated between environmental conditions and interpreted as a systemic pro/anti-inflammatory index (Shamsi et al. 2017).

Complementary to the previous comparison tests, the magnitude of the changes was quantified using the standardized differences based on Cohen’s effect sizes (ES). These measures were calculated as the mean change between measurements (HH-N; or post-exercise – pre-exercise absolute peak value) divided by the pooled SD for all pairs of comparisons. The thresholds for interpretation of Cohen’s d were set as follows: < 0.2 (trivial), 0.21–0.5 (small), 0.5–0.8 (moderate), 0.8–1.3 (large), and > 1.30 (very large) (Juandi, et al. 2021).

The impact of environmental conditions on predicting the inflammation response from metabolites, myokines, and TAC during the training program was estimated through multiple linear regression models using a manual stepwise approach. When appropriate, residual standard errors (RSE) and coefficient of determination (R^2^) were used to assess prediction models. Those variables with a risk of multicollinearity and potential confounding were excluded from the models. Due to the high correlation between the “*condition”* variable with both IL-10 and HSP70 (Supplemental Table 2), separate regression analyses were subsequently conducted within HH and N conditions.

All data extraction and analyses were performed using R Statistics version 2023.06.1 + 524 (R.D.C., R 2010) and IBM SPSS Statistics version 28.0.1.0 for MacOS (IBM Corp., Armonk, NY, USA). The level of significance was set at *p* < 0.05.

## Results

**Fig. 1:**
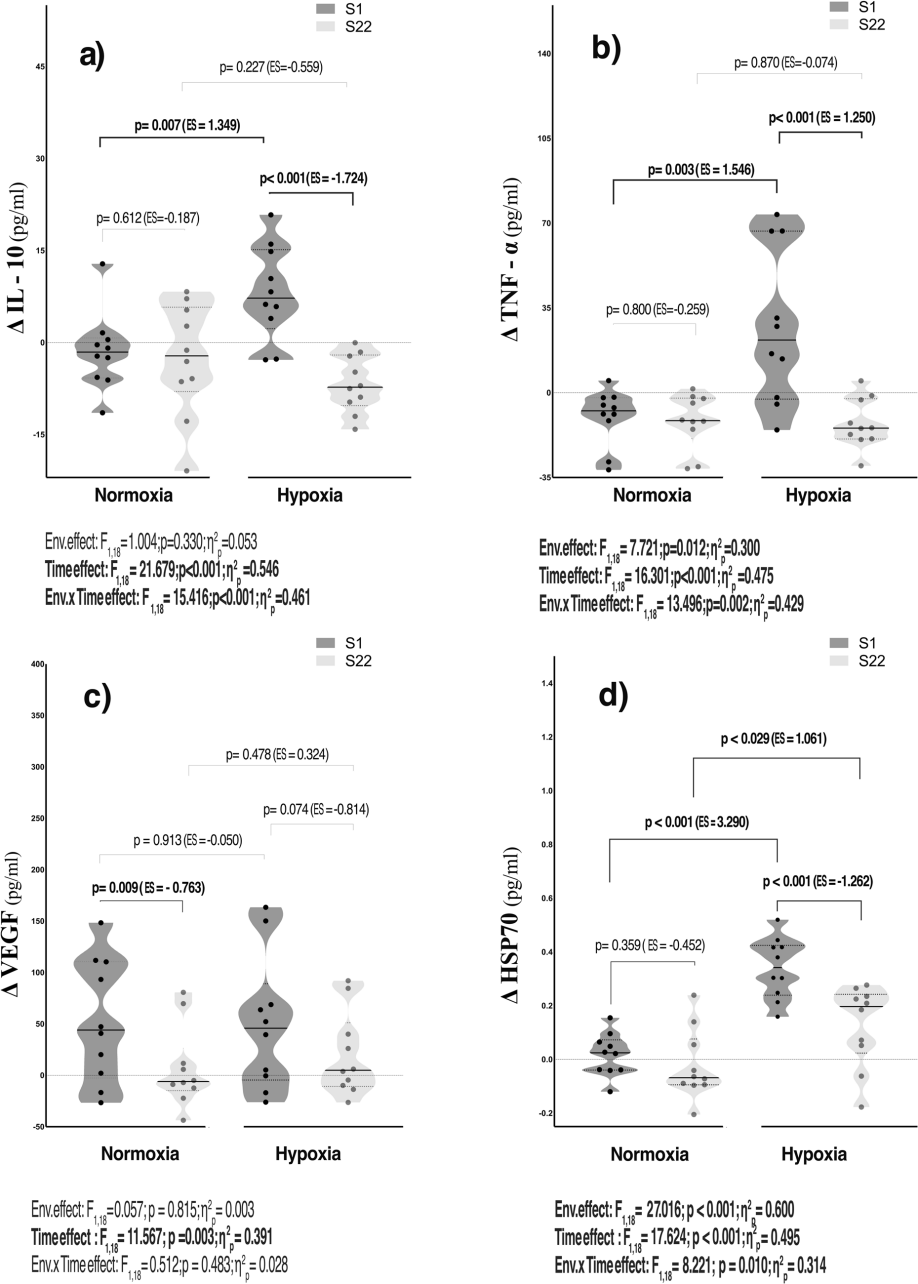
Violin plots for analysis of circulating cytokines IL-10 (**a**), TNF-α (**b**), VEGF (**c**), and HSP70 (**d**) response throughout the recovery before and after the R_T_ program. The black line represents the respective group median (central line) and interquartile range (lower and upper dash lines). Dots represent single-subject data corresponding to the delta change (Δ) between the peak values of the training session recovery and the baseline condition established 72 h before the resistance training program. *p* value (*p* < 0.05), partial eta squared from ANOVA (η^2^_p_), Cohen’s d effect size (ES). ES was calculated as (Δ the peak value of post-exercise–pre-exercise of S22 vs. S1) or (HH-N) divided by the pooled standard deviation

The comparison of cytokines change scores between conditions following an 8-week R_T_ program is presented in Fig. 1a–d. Serum IL-10 displayed a large time (*p* < 0.001; η^2^_p_ = 0.546) and environmental x time effects (*p* < 0.001; η^2^_p_ = 0.461) after the training. Compared with N, the HH group yielded a very large increase of IL-10 at S1 (ES = 1.349; *p* = 0.007). Nevertheless, a very large reduction after the R_T_ program was only found in HH when compared with the baseline (ES = − 1.724; *p* < 0.001) (Fig. 1a). Circulating TNF-α revealed a large environmental (*p* = 0.012; η^2^_p_ = 0.300), time (*p* < 0.001; η^2^_p_ = 0.475), and environmental x time effects (*p* = 0.002; η^2^_p_ = 0.429) (Fig. 1b). Similar to the IL-10 trend, pairwise comparison exhibited a very large increase of TNF-α in HH at S1 compared with the N condition (ES = 1.546; *p* = 0.003). Conversely, a large reduction was found only in HH following the R_T_ program (ES = -1.250; *p* < 0.001) (Fig. 1b). A similar large time effect (*p* = 0.003; η^2^_p_ = 0.391) was found in VEGF along the R_T_ program. Circulating HSP70 exhibited a large environmental (*p* < 0.001; η^2^_p_ = 0.600), time (*p* < 0.001; η^2^_p_ = 0.495), and environmental x time effects (*p* = 0.010; η^2^_p_ = 0.314) (Fig. 1-d). Compared with N, the HH group revealed a large to very large increase in HSP70 release either at S1 (ES = 3.290; *p* < 0.001) and S22 (ES = 1.061; *p* < 0.029).

**Fig. 2:**
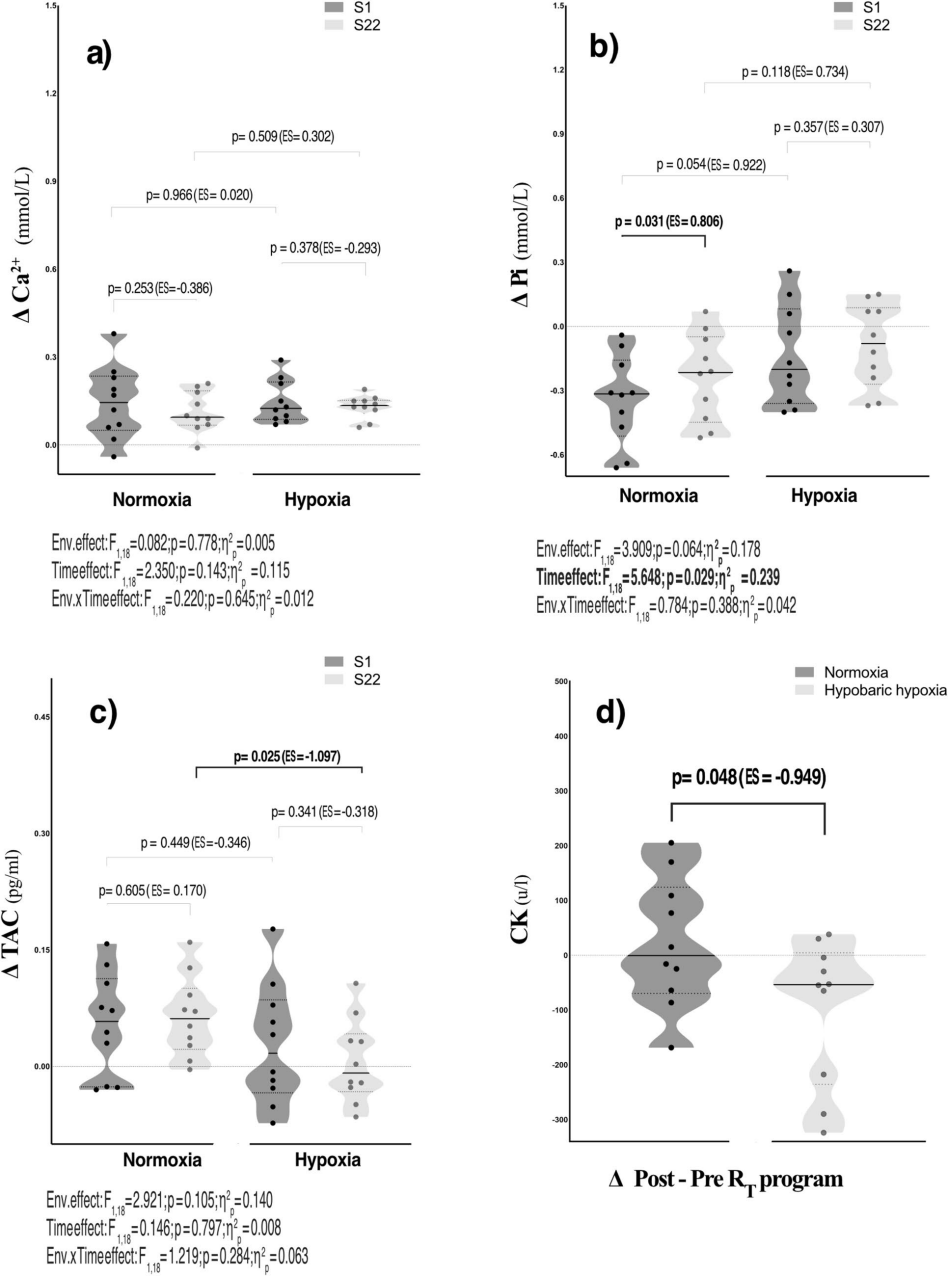
Violin plots for analysis of circulating cytokines, calcium (**a**), inorganic phosphate (**b**), total antioxidant capacity (**c**), and creatine kinase (**d**) response throughout the recovery before and after the R_T_ program. The black line represents the respective group median (central line) and interquartile range (lower and upper dash lines). Dots represent single-subject data corresponding to the delta change (Δ) between the peak values of the training session recovery and the baseline condition established 72 h before the resistance training program. *p*-value (*p* < 0.05), partial eta squared from ANOVA (η^2^_p_), Cohen’s d effect size (ES). ES was calculated as (Δ peak value of post-exercise–pre-exercise of S22 vs. S1) or (HH-N) divided by the pooled standard deviation. Ca^2+^: calcium; CK: creatine kinase; Pi: inorganic phosphate; TAC: total antioxidant capacity

Figure 2a–d displays the effects of the R_T_ program on antioxidants, muscle damage, and metabolic stress variables in different conditions. Serum Pi only exhibited a time effect after the R_T_ program (*p* = 0.029; η^2^_p_ = 0.239). However, pairwise comparison tests between conditions did not reach statistical significance for either circulating Ca^2+^ and Pi levels (all *p* > 0.05) (Fig. 2 a, b). Conversely, compared with the N condition, serum TAC (ES = − 1.097; *p* = 0.025) (Fig. 2-c) and CK (ES = -0.949; *p* = 0.048) (Fig. 2d) were significantly reduced in HH at S22.

Multiple linear regression models predicting circulating TNF-α levels in the HH condition revealed that serum HSP70 and IL-10 reached to explained ~ 66% of the variance (Table 1). No significant changes were observed in the N group (Table 2).

**Table 1:** Multiple linear regression models predicting circulating TNF-α levels in HH conditions

Multiple linear regression models predicting circulating TNF-α levels in HH conditions				
	Model 1		Model 2	
	Estimate (CI)	Std. error	Estimate (CI)	Std. error
(Constant)	− 11.48 (− 40.62, 17.66)	12.64	23.44 (− 17.16, 64.05)	17.17
HSP70 (pg/ml)	134.18* (24.61, 243.74)	47.51	165.65** (73.08, 258.23)	39.15
IL-10 (pg/ml)			1.90* (0.09, 3.70)	0.76
n	20		20	
R^2^/ adj. R^2^	0.50/0.44		0.73/ 0.66	
F-statistic	7.97*		9.64**	

*HH* hypobaric hypoxia; *HSP70* heat shock protein 70; *IL-10* interleukin 10; *pg/ml* picograms per milliliter; *R*^*2*^ coefficient of determination; Std.error standard error; *TNF-α* tumor necrosis factor alpha; 95% confidence interval (CI)

*p < 0.05

**p < 0.01

Bold indicates statistically significant coefficients

**Table 2:** Multiple linear regression models predicting circulating TNF-α levels in N conditions

Multiple linear regression models predicting circulating TNF-α levels in N conditions				
	Model 1		Model 2	
	Estimate (CI)	Std. error	Estimate (CI)	Std. error
(Constant)	− 0.44 (− 6.05, 5.16)	2.43	− 1.31 (− 7.18, 4.56)	2.48
HSP70 (pg/ml)	36.18 (− 24.15, 96.39)	26.14	27.42 (− 35.35, 90.20)	26.55
IL-10 (pg/ml)			− 0.41 (− 1.23, 0.41)	0.35
*n*	20		20	
R^2^/ adj. R^2^	0.19/ 0.09		0.33/ 0.13	
F-statistic	1.91		1.70	

*HSP70* heat shock protein 70; *IL-10* interleukin 10; *N* normoxia; *pg/ml* picograms per milliliter; *R*^*2*^ coefficient of determination; Std.error standard error; *TNF-α* tumor necrosis factor alpha; 95% confidence interval (CI)

*p < 0.05

**p < 0.01

Furthermore, compared with N, both the 1RM-SQ (N_pre-RT_: 1.33 ± 0.13, N_post-RT_: 1.51 ± 0.20 and HH_pre-RT_: 1.11 ± 0.24, HH_post-RT_: 1.42 ± 0.21 kg·kg^−1^bw) and SJ (N_pre-RT_: 30.03 ± 5.84, N_post-RT_: 27.66 ± 3.30 and HH_pre-RT_: 23.26 ± 4.14, HH_post-RT_: 27.79 ± 3.35 cm) exhibited large to very large increases favoring the HH condition after the R_T_ program in 1RM-SQ (ΔHH: 0.22 ± 0.07 vs. ΔN: 0.14 ± 0.09 kg·kg^−1^bw; ES = 0.97; *p* = 0.044) and SJ (ΔHH: 4.52 ± 2.56 vs. ΔN: -2.37 ± 3.92 cm; ES = 2.08; *p* < 0.001) absolute delta changes outcomes.

## Discussion

Results reveal that chronic intermittent HH exposure during an 8-week R_T_ program may reflect variations in immune-inflammatory and muscle remodeling regulatory mechanisms in young males, thereby mitigating muscle damage and preserving muscle quality. This adaptation appears to elicit greater improvements in power and maximal muscle contractile capacity than the same intervention in N. To our knowledge, this is the first study that analyses the cumulative effects of terrestrial intermittent HH during long-term R_T_ protocols on systemic inflammatory biomarkers.

A prolonged low-grade chronic inflammation is characterized by impaired mitochondrial function and anabolic resistance processes (Kunz and Lanza 2023). However, it is well established that acute bouts of exercise (e.g., high volumes and/or intensities, eccentric contractions, and prolonged exercise) induce inflammatory responses that may exhibit several benefits, such as muscle repair and regeneration after injury or exercise (Methenitis et al. 2021). In addition, Perez-Regalado et al. (Pérez-Regalado 2023) reported increased circulating inflammatory cytokines following acute resistance exercise at moderate HH, accompanied by a compensatory anti-inflammatory response, ensuring the pro- and anti-inflammatory balance. Consistent with these studies (Pérez-Regalado 2023; Khalafi et al. 2023b; Tsukui et al. 2000), R_T_ in HH depicted a substantially bigger increase in circulating TNF-α, which returned to baseline throughout the 8-week R_T_ program, than N (Fig. 1b). This response is essential for the activation, differentiation, and proliferation of muscle satellite cells during the early stages of tissue regeneration after exercise-induced damage (Ji et al. 2022). Accordingly, our results showed a similar trend in serum IL-10 in both conditions (Fig. 1a, Supplemental Table 1), supporting the pro- and anti-inflammatory balance (Shamsi et al. 2017). Remarkably, IL-10 is predominantly recognized for its pivotal role in inhibiting the expression of TNF-α and interferon-gamma, both considered the main contributors to pro-inflammatory and cytotoxic responses via M1 macrophages. IL-10 also governs the shift from M1 to M2 macrophage phenotype (Cornish et al. 2020). This process involves several adaptive responses aimed at regulating muscle tissue repair and regeneration, as well as maintaining skeletal muscle homeostasis (Rogeri et al. 2020). Both factors ensure skeletal muscle health and strength production, particularly when combining R_T_ periods with demanding environmental conditions like the HH. The low concentration of circulating IL-10 at the end of the 8 weeks of R_T_ in HH (Fig. 1a) indicates no major ongoing pro-inflammatory activity, while suggesting the proper assimilation of the higher stress induced by the HH condition. This finding is consistent with previous research (Siqueira et al. 2018; Tibana et al. 2019) supporting the crucial role of a balanced inflammatory status as a critical determinant for enhancing recovery.

Circulating HSP70 is upregulated in response to low-oxygen environments, a process mediated by the activation of HIF1-α (Cao et al. 2021). HIF1-α positively regulates VEGF expression (Ohno et al. 2012), an inducer of endothelial cell proliferation, while facilitating muscle regeneration and mitigating fibrosis (Frey, et al. 2012). Surprisingly, serum VEGF measured at the end of the R_T_ under HH in our study evidenced no change with respect to the baseline and/or between conditions (Fig. 1c). Other research stated the challenge of detecting any significant peripheral (Maga et al. 2023; Song 2023; Gunga et al. 1999) or intracellular (Larkin et al. 2012) changes in VEGF, suggesting a possible alteration in the removal and/or binding processes. Although it remains unclear whether circulating HSP70 affects the VEGF concentration in chronic intermittent hypoxia exposure, HSP70 appears to downregulate HIF1-α levels via selective ubiquitination (Luo et al. 2010). Consequently, the rapid increase in circulating HSP70 during the R_T_ program in HH likely mitigates excessive inflammatory responses. This effect is achieved by facilitating the degradation of HIF1-α, which is linked to the upregulation of several inflammatory pathways following tissue injury (Sadiku and Walmsley 2019). Accordingly, compared with N, HH reported a marked increase in this cytokine following the entire R_T_ period condition (Fig. 1d). In addition, HSP70 is widely recognized for its immunosuppressive capacity (Borges et al. 2012), acting as a cytoprotective stress sensor, primarily by preventing cells from apoptosis (Schett et al. 2003). HSP70 inhibits TNF-α-mediated inflammatory pathways while promoting cell survival via IL-10 upregulation (Ferat-Osorio et al. 2014; Wachstein et al. 2012). Consistently, circulating HSP70 predicted a marked reduction in TNF-α, reaching to explain ~ 44% of the variance (Table 1). In this sense, it could be presumed that the interplay between the exacerbated HSP70 response under intermittent HH conditions and the R_T_ aimed to mitigate the negative effects of chronic TNF-α-induced inflammatory response on muscle mass regeneration (see Tables 1–2). This step is crucial, as the anti-inflammatory response follows the initial inflammatory phase, ensuring the capacity of satellite cells to differentiate efficiently into mature muscle fibers. Thereby, this process facilitates muscle tissue regeneration and the restoration of functional integrity (Ji et al. 2022).

Finally, it is noteworthy that no changes of interest were found in the acute response of the metabolic stress variables, such as serum Pi and Ca^2+^, after the program’s first and last R_T_ sessions. According to previous research, the lack of evidence could be due to the time window kept from the end of the exercise to the first blood draw (5 min), during which the rapid recapture of both metabolites occurs, facilitating the restoration of normal cellular energy production processes in the early stages of recovery (Pérez-Regalado 2023; Feriche et al. 2020). In accordance with our results, Krüger et al. (2019) proposed that regular exercise decreases plasma CK through a HSP70-dependent mechanism, thereby preventing muscle damage. These findings could also explain the reduced circulating TAC found in HH (Fig. 2c). The literature has already described a relationship between the increase of pro-oxidants and TAC as body redox potential, following exercise interventions (Sotler, et al. 2019; Azizbeigi et al. 2014; Nanakorn and Chuechan 2022). Research suggests that the absence of increased TAC could be due to physiological adaptations from repetitive stress-induced exercise or environment, leading to the upregulation of other independent molecular pathways (e.g., superoxide dismutase enzyme, among others). In this context, the decrease observed in serum CK and TAC levels throughout the R_T_ in the HH group reinforces the notion of proper adaptation to training loads applied within an environment of added stress induced by hypoxia (Azizbeigi et al. 2014). It can be assumed that restoring circulating inflammatory biomarkers (Alloatti et al. 2000) and CK levels, even below the recommended clinical values (~ 200 u/l) (Tibana et al. 2019; Chatzinikolaou et al. 2010), may also positively enhanced skeletal muscle contractility and explosive capacity. However, these results should be interpreted with caution.

Our findings align with a recent meta-analysis (Benavente and Feriche 2024) that indicated a slight benefit of R_T_ under HH over N for muscle strength gains. Although in our study both groups experienced gains in maximal strength, the increase was greater in the HH group (*p* = 0.041; ES = 0.99), as also happened in SJ (*p* = 0.001; ES = 2.0). However, no significant differences in SJ were observed in the N group, indicating that pre- and post-R_T_ values remained unchanged. In addition, since the R_T_ program did not include jump execution, no significant improvements in SJ were expected (Marshall et al. 2021). This hypothesis is in accordance with other studies on this topic (Benavente 2024; Pérez-Regalado et al. 2024; Márquez et al. 2023; Inness, et al. 2016). Reducing oxygen availability might affect central and peripheral nervous system functions through the carotid oxygen sensor-induced responses (Burtscher et al. 2022). This potentially heightened excitability may facilitate certain neural adaptations, particularly after physical activities like resistance exercise, by increasing the responsiveness of motor pathways and reaction times. The combination of these factors positively influences both the contractile and explosive capacity in HH to a greater extent than the N group.

This research has some limitations that should be noted: (1) the sample size was relatively small, which could influence the width of probability distributions across the outcomes. Logistical constraints and the cost of biomarker analyses limited the recruitment of a larger sample. However, results from this population are of specific interest; (2) blood samples were collected exclusively within the 5–30 min post-exercise window which limits our ability to draw conclusions beyond this acute time frame; and (3) the findings are specific to the studied population, which may restrict generalizability to broader groups.

In summary, our findings have provided solid evidence that the attenuation of TNF-α throughout a R_T_ period under intermittent HH conditions in young, trained males may be attributed to the systemic HSP70 response. This could be considered a regulatory mechanism for mitigating muscle damage and controlling the secretion of pro- and anti-inflammatory cytokines (e.g., IL-10), preserving muscle quality. In addition, 8 weeks of R_T_ in HH substantially improved contractile and explosive capacity more than N. Overall, these findings contribute to elucidating the use of moderate intermittent HH with long-term R_T_ interventions to enhance overall stress while maintaining a pro- and anti-inflammatory balance essential for skeletal muscle adaptations.

## Supplementary Information

Below is the link to the electronic supplementary material.

Supplementary file1 (DOCX 15 KB)

Supplementary file2 (DOCX 19 KB)

## Data Availability

The data that support the findings of this study are available from the corresponding author upon reasonable request.
