# Supplementary material for: New insights into the integrative effects of resistance training at moderate altitude on systemic inflammation
Source: Eur J Appl Physiol. 2025 Jun 7;125(11):3311–21. doi: 10.1007/s00421-025-05842-x (PMC12528297; doi:10.1007/s00421-025-05842-x)
Supplement: Supplementary file 1 — Supplementary file1 (DOCX 15 KB) [file 421_2025_5842_MOESM1_ESM.docx]

|  |  | **N** (means ± SD) | **HH** (means ± SD) | **HH vs. N**  *p* value (ES) |
| --- | --- | --- | --- | --- |
| **IL-10/ TNF-α ratio** | Δ S1 | -0.140 ± 0.821 | -0.242 ± 1.272 | 0.833 (-0.096) |
|  | Δ S22 | -0.602 ± 1.619 | 0.389 ± 0.763 | 0.097 (0.783) |
|  | Δ S22 - S1 | -1.635 ± 7.505 | -0.529 ± 2.234 | 0.661 (0.200) |

P-value (p < 0.05); IL-10: interleukin 10; TNF-α: tumor necrosis factor alpha; SD: standard deviation; cm: centimetres; Cohen’s d effect size (ES).
