# Supplementary material for: New insights into the integrative effects of resistance training at moderate altitude on systemic inflammation
Source: Eur J Appl Physiol. 2025 Jun 7;125(11):3311–21. doi: 10.1007/s00421-025-05842-x (PMC12528297; doi:10.1007/s00421-025-05842-x)
Supplement: Supplementary file 2 — Supplementary file2 (DOCX 19 KB) [file 421_2025_5842_MOESM2_ESM.docx]

|  |  | **Environmental condition** | | | | | |  |
| --- | --- | --- | --- | --- | --- | --- | --- | --- |
|  |  | **Estimate (CI)** | | **Std. error** | **n** | **R^2^/ adj. R^2^** | **F-statistic** | |
| **Multiple linear regression models predicting** | ***Model 1***  **HSP70** (pg/ml) | | **-0.173**** (-0.299, -0.046) | 0.060 | 20 | 0.314/ 0.275 | **8.221**** | |
|  | Constant | | 0.133 (-0.067, 0.332) | 0.095 |  |  |  |  |
|  | ***Model 2***  **TNF-α** (pg/ml) | | **-38.116**** (-59.914, -16.318) | 10.375 | 20 | 0.429/ 0.397 | **13.496**** | |
|  | Constant | | **36.229*** (1.764, 70.695) | 16.405 |  |  |  |  |
|  | ***Model 3***  **IL-10** (pg/ml) | | **-13.617***** (-20.904, -6.331) | 3.468 | 20 | 0.461/ 0.431 | **15.416***** | |
|  | Constant | | **12.352*** (0.831, 23.873) | 5.484 |  |  |  |  |

95% confidence interval (CI); * p < 0.05, ** p < 0.01, *** p < 0.001.
